# Lipopolysaccharide (LPS) Promotes Apoptosis in Human Breast Epithelial × Breast Cancer Hybrids, but Not in Parental Cells

**DOI:** 10.1371/journal.pone.0148438

**Published:** 2016-02-10

**Authors:** Sabrina Fried, Songuel Tosun, Gabriele Troost, Silvia Keil, Kurt S. Zaenker, Thomas Dittmar

**Affiliations:** Institute of Immunology & Experimental Oncology, Center for Biomedical Education and Research, Witten/Herdecke University, Stockumer Str. 10, Witten, Germany; Chang-Gung University, TAIWAN

## Abstract

Toll-like receptors (TLRs) belong to the group of pathogen recognition receptors known to play a crucial role in the innate immune system. In cancer, TLR expression is still debated controversially due to contradictory results reporting that both induction of apoptosis as well as tumor progression could depend on TLR signaling, whereby recent data rather indicate a pro-tumorigenic effect. The biological phenomenon of cell fusion has been associated with cancer progression due to findings revealing that fusion-derived hybrid cells could exhibit properties like an increased metastatogenic capacity and an increased drug resistance. Thus, M13MDA435 hybrid cell lines, which derived from spontaneous fusion events between human M13SV1-EGFP-Neo breast epithelial cells and human MDA-MB-435-Hyg breast cancer cells, were investigated. Cultivation of cells in the presence of the TLR4 ligand LPS potently induced apoptosis in all hybrid clones, but not in parental cells, which was most likely attributed to differential kinetics of the TLR4 signal transduction cascade. Activation of this pathway concomitant with NF-**κ**B nuclear translocation and TNF-**α** expression was solely observed in hybrid cells. However, induction of LPS mediated apoptosis was not TNF-**α** dependent since TNF-**α** neutralization was not correlated to a decreased amount of dead cells. In addition to TNF-**α**, LPS also caused IFN-**β** expression in hybrid clones 1 and 3. Interestingly, hybrid clones differ in the mode of LPS induced apoptosis. While neutralization of IFN-**β** was sufficient to impair the LPS induced apoptosis in M13MDA435-1 and -3 hybrids, the amount of apoptotic M13MDA435-2 and -4 hybrid cells remained unchanged in the presence of neutralizing IFN-**β** antibodies. In summary, the fusion of non-LPS susceptible parental human breast epithelial cells and human breast cancer cells gave rise to LPS susceptible hybrid cells, which is in view with the cell fusion hypothesis that hybrid cells could exhibit novel properties.

## Introduction

The role of Toll-like receptors (TLRs) in cancer is still debated controversially due to contradictory results reporting that both induction of apoptosis as well as tumor progression could depend on TLR signaling (for review see: [1–3]). On the one hand, various data demonstrated the impact of TLR signaling in suppressing cancer growth. For instance, superficial forms of bladder cancer were effectively treated with the *Mycobacterium bovis* bacillus Calmette-Guérin (BCG) vaccine for more than 30 years [3, 4]. Likewise, an anti-tumorigenic effect has also been suggested for the TLR4 agonist lipopolysaccharide (LPS; from Gram-negative bacteria) for the treatment of colorectal cancer and glioblastoma multiforme [5, 6]. In addition to LPS, CpG oligodeoxynucleotides (CpG-ODN) binding to TLR9 have also been tested for the treatment of glioblastoma patients, but data indicate that only some glioblastoma multiforme patients might benefit from this therapy [7].

In contrast to the putative anti-tumorigenic effects of TLR-signaling an increasing body of evidence rather points to a TLR-dependent tumor progression. Administration of endotoxin/LPS was correlated to an increased lung metastasis in a murine mammary cancer model of metastatic disease, which was attributed to an increased proliferation and decreased rate of apoptosis of tumor cells [8] as well as an increased angiogenesis, vascular permeability and tumor cell invasion and migration [9]. Yang and colleagues demonstrated that LPS triggered the metastatic spreading of human MDA-MB-231 breast cancer into liver of nude mice in a TLR4-dependent manner [10], which may also depend on a TLR4-dependent **α**_v_**β**_3_-mediated adhesion of metastatic breast tumor cells to the endothelial lining [11]. These findings are in view with data of Hsu et al. revealing that the LPS-induced TLR4 signaling in human colorectal cancer cells increased the β_1_-integrin-mediated cell adhesion and liver metastasis formation [12]. Clinicopathological parameters further revealed that TLR4 overexpression in human breast cancer tissues was correlated to lymph node metastasis [10]. In a recent work Volk-Draper et al. demonstrated that the chemotherapeutic compound paclitaxel binds to TLR4, thereby inducing a specific TLR4 signaling resulting in the expression of inflammatory mediators that promote angiogenesis, lymphangiogenesis and metastasis both at local sites and premetastatic niches [13]. Similar findings were reported for TLR4 signaling and paclitaxel chemoresistance in ovarian cancer [14] suggesting a putative role of TLR4 signaling in the development of chemoresistant cancer cells.

The biological phenomenon of cell fusion plays a crucial role in various physiological events, like fertilization, placentation, tissue regeneration/wound healing, as well as pathophysiological events including cancer (for review see: [15, 16]). Particularly in the context of cancer it is assumed that fusion events between tumor cells and tumor cells [17, 18] as well as tumor cells and normal cells, like macrophages [19, 20], fibroblasts [21] or epithelial cells [22–24], could give rise to hybrid cells exhibiting novel properties, such as an enhanced metastatic capacity, drug resistance or an increased resistance to undergo apoptosis [25–29]. Spontaneous fusion was observed between acute leukemia cells and macrophages and has been suggested as a mechanism of gene transfer for cancer dissemination and hybrid cell mediated perpetuation of leukemia [30]. In human and mouse epithelial ovarian carcinoma the interaction between tumor infiltrating hematopoietic cells and cancer cells resulted in fusion-derived hybrid cells expressing various hematopoietic lineage markers including CD45 [31]. Of interest were findings indicating an increased CXCR4 expression in hybrid cells, which may be related to metastatic spreading [31]. This would be in view with recent data indicating that fusion between gastric epithelial cells and mesenchymal stem cells resulted in epithelial-to-mesenchymal and malignant transformation of the hybrid cells [32].

Because of the plethora of data indicating that both TLR signaling and cell fusion have been associated with tumor progression we decided to investigate TLR expression and signaling in M13MDA435 hybrid cell lines, which originated from spontaneous fusion events between human M13SV1-EGFP-Neo breast epithelial cells exhibiting stem cell properties and human MDA-MB-435-Hyg breast cancer cells [23, 33]. We have already demonstrated that these hybrid cell lines responded to EGF with an increased migratory activity and exhibited a differential RAF-AKT crosstalk [24] assuming that these cells will responding differently in comparison to their parental cells. Interestingly, M13MDA435 hybrid cell lines truly showed a differential, but rather an unexpected TLR4 signaling since LPS potently induced apoptosis in all hybrid cell lines, but not in the parental cells.

## Materials and Methods

### Cell culture

All cell lines were cultivated at 37°C and 5% CO_2_ in a humidified atmosphere as described previously [23, 24]. In brief, M13SV1-EGFP-Neo human breast epithelial cells exhibiting stem-like properties were maintained in MSU-1 basal media (Biochrom GmbH, Berlin, Germany) supplemented with 10% fetal calf serum (FCS) (Biochrom GmbH, Berlin, Germany), 1% Penicillin/ Streptomycin (100U/ml Penicillin, 0.1mg/ml Streptomycin; Sigma Aldrich, Taufkirchen, Germany), 10**μ**g/ml human recombinant EGF, 5**μ**g/ml human recombinant Insulin, 0.5**μ**g/ml Hydrocortisone, 4**μ**g/ml human Transferrin, 10nM **β**-Estrogen (all chemicals were purchased from Sigma Aldrich, Taufkirchen, Germany), and 400**μ**g/ml G418 (Biochrom GmbH, Berlin, Germany). M13SV1-EGFP-Neo cells were generated by stable transfection of the M13SV1 human breast epithelial cells (kindly provided by James E. Trosko (Michigan State University, East Lansing, MI [34]) with the EGFP-MCS-Neo vector containing an EGFP expression cassette and a neomycin/G418 resistance [35]. The breast cancer cell line MDA-MB-435-Hyg was cultivated in DMEM (Sigma Aldrich, Taufkirchen, Germany) supplemented with 10% FCS (Biochrom GmbH, Berlin, Germany), 1% Penicillin/ Streptomycin (Sigma Aldrich, Taufkirchen, Germany), and 200**μ**g/ml Hygromycin B (Pan-Biotech GmbH, Aidenbach, Germany). MDA-MB-435-Hyg cells were generated by stable transfection of human MDA-MB-435 breast cancer cell line (HTB 129; LGC Standards GmbH, Wesel, Germany) with the pKS-Hyg vector. M13MDA435-X (X = 1–4) hybrid cell clones were cultivated in DMEM (Sigma Aldrich, Taufkirchen, Germany) supplemented with 10% FCS (Biochrom GmbH, Berlin, Germany), 1% Penicillin/ Streptomycin (Sigma Aldrich, Taufkirchen, Germany), 400**μ**g/ml G418 (Biochrom GmbH, Berlin, Germany) and 200**μ**g/ml Hygromycin B (Pan-Biotech GmbH, Aidenbach, Germany). All hybrid cell clones were derived from spontaneous fusion events between M13SV1-EGFP-Neo and MDA-MB-435-Hyg cells [23, 33].

### Antibodies, reagents, inhibitors

Anti-histone H3 ChIP grade (rabbit polyclonal; Abcam, Cambridge, UK), anti-NF-**κ**B p65 (rabbit polyclonal; Santa Cruz Biotech, Heidelberg, Germany), anti-Myd88 (rabbit monoclonal; Cell Signaling, Frankfurt/Main, Germany), anti-TLR2 (rabbit polyclonal; Santa Cruz Biotech, Heidelberg, Germany), anti-TLR3 (rabbit polyclonal; Abgent Inc., San Diego, CA; USA), anti-TLR4 (rabbit polyclonal; Santa Cruz Biotech, Heidelberg, Germany), anti-TLR5 (rabbit polyclonal; Antibodies-online GmbH; Aachen, Germany), anti-TLR9 (rabbit polyclonal; ProSci Inc., Poway, CA; USA), anti-TNFR1 (rabbit monoclonal; Cell Signaling, Frankfurt/Main, Germany), anti-TNFR2 (rabbit polyclonal; Cell Signaling, Frankfurt/Main, Germany), anti-TRAF6 (rabbit monoclonal; Cell Signaling, Frankfurt/Main, Germany), anti-TRIF (rabbit polyclonal; Cell Signaling, Frankfurt/Main, Germany), anti-IFNAR1 (rabbit monoclonal; Abcam, Cambridge, UK), anti-TNF-**α** (neutralization: mouse monoclonal; Abcam, Cambridge, UK; Western Blot: rabbit monoclonal; Cell Signaling, Frankfurt/Main, Germany), anti-IFN-**β** (mouse monoclonal; Biozol GmbH, Eching, Germany), anti-**β**-actin (rabbit monoclonal; Cell Signaling, Frankfurt/Main, Germany), anti-elf4E (rabbit polyclonal; Cell Signaling, Frankfurt/Main, Germany), anti-mouse-IgG-HRP-linked (Cell Signaling, Frankfurt/Main, Germany), anti-rabbit-IgG-HRP-linked (Cell Signaling, Frankfurt/Main, Germany), Lipopolysaccharide (LPS) (from *E*. *Coli*; Sigma Aldrich, Taufkirchen, Germany), Flagellin (from *Salmonella Typhimurium*; Biomol GmbH, Hamburg, Germany), caspase-8 inhibitor (Z-IETD-FMK; R&D Systems, Wiesbaden-Nordenstedt, Germany), caspase-10 inhibitor (Z-AEVD-FMK; R&D Systems, Wiesbaden-Nordenstedt, Germany).

### XTT proliferation assay

XTT cell proliferation assay was performed as described previously [35, 36]. In brief, cells (5×10^3^/ well) were seeded in triplicates in a 96-well flat-bottom microtiter plate in 0.1ml of the appropriate culture medium containing different concentrations (50ng/ml, 100ng/ml, 150ng/ml) of LPS and Flagellin, respectively. Non-treated cells served as a control. After 24h, 48h, 72h, and 96h media was removed and plates were analyzed with XTT reagent (Roche Diagnostics, Mannheim, Germany) according to the manufacturer’s instructions. The absorption of the formed XTT-formazan derivative was measured using a BioTek EL800 microplate reader (BioTek, Bad Friedrichshall, Germany). The EGFP fluorescence of M13SV1-EGFP-Neo cells and M13MDA435-1 and -3 hybrids did not interfere with the XTT-formazan formed derivative.

### RT-PCR

RNA was isolated from 1×10^6^ cells by using the NucleoSpin^®^ RNA II Kit from Macherey-Nagel (Macherey-Nagel GmbH, Düren, Germany) in accordance to the manufacturers’ instructions. Reverse Transcription of RNA into cDNA was performed using the RevertAid^™^ First Strand cDNA Synthesis Kit (VWR International, Darmstadt, Germany) as referred to the instruction manual. PCR was performed in a 25**μ**l reaction mixture containing 1.25U Taq Polymerase, 1× reaction buffer, 2mM MgCl2, 200**μ**M of each dNTP (all reagents were purchased from VWR International, Darmstadt, Germany) and 100**μ**M primers (Life Technologies, Darmstadt, Germany). The cycling conditions comprised of an initial denaturation of 5min at 94°C and 30 cycles of 0.5min at 94°C, 0.5min at the appropriate annealing temperature and 0.5min at 72°C followed by a final elongation for 10min at 72°C. All used primer pairs concomitant with their specific annealing temperature and product length are summarized in Table 1. PCR products were separated on a 1% agarose gel. Bands were visualized by GelRed^™^ staining (VWR International GmbH, Darmstadt, Germany) and the GelDoc^™^ EZ Imager system (Bio-Rad, Munich, Germany).

**Table 1:** Summary of PCR primer pairs.

Name	Annealing Temperature (°C)	Mean product size	Primer	Sequence (5’ to 3’)
**β**-actin	55	290	forward	GTGACGTTGACATCCGTAAAGACC
			reverse	TCAGTAACAGTCCGCCTAGAAGCA
FasL	55	231	forward	CTGGGGATGTTTCAGCTCTTC
			reverse	CTTCACTCCAGAAAGCAGGAC
FasR	60	384	forward	ATGCTGGGCATCTGGACCCTCCTA
			reverse	TCTGCACTTGGTATTCTGGGTCCG
IFN-**β**	55	174	forward	AGTAGGCGACACTGTTCGTG
			reverse	AGCCTCCCATTCAATTGCCA
IL-1**β**	55	219	forward	AGCCATGGCAGAAGTACCTG
			reverse	TCCATGGCCACAACAACTGA
TLR1*	55	517	forward	TCTGGTACACGCATGGTC
			reverse	ATGGGTGGGAAACTGAAT
TLR2*	55	264	forward	AACTTACTGGGAAATCCTTAC
			reverse	AAAAATCTCCAGCAGTAAAAT
TLR3*	55	131	forward	GCATTTGTTTTCTCACTCTTT
			reverse	TTAGCCACTGAAAAGAAAAAT
TLR4*	55	106	forward	CGAGGAAGAGAAGACACCAGT
			reverse	CATCATCCTCACTGCTTCTGT
TLR5*	55	383	forward	AGCTTCAACTATATCAGGACA
			reverse	TGGTTGGAGGAAAAATCTAT
TLR6*	55	123	forward	CTTCCATTTTGTTTGCCTTAT
			reverse	AGCGGTAGGTCTTTTGGAAC
TLR7*	55	149	forward	AAACTCCTTGGGGCTAGATG
			reverse	AGGGTGAGGTTCGTGGTGTT
TLR8*	55	246	forward	CTGTGAGTTATGCGCCGAAGA
			reverse	TGGTGCTGTACATTGGGGTTG
TLR9	55	242	forward	GTTGCAAGGCTGTGGTGAAG
			reverse	CTGGATAGCACCAGTAGCGG
TLR10*	55	279	forward	AGAAGAAAGGGAACTGATGAC
			reverse	CCTGCCAGTAAATACCAAGT
TNF-**α**	55	109	forward	AACATCCAACCTTCCCAAACG
			reverse	GACCCTAAGCCCCCAATTCTC

* primer sequences were chosen from [37]

### Flow cytometry

Flow cytometry was performed using a FACScalibur flow cytometer (Becton Dickenson, Heidelberg, Germany). Apoptosis measurements cells were performed by using the Alexa Fluor^®^ 488Annexin V/Dead Cell Apoptosis Kit (Life Technologies, Darmstadt, Germany). Cells (2×10^5^) were cultivated in duplicates in a 6-well plate at 37°C and 5% CO_2_ in a humidified atmosphere. Two hours after seeding supplements of interest were added. After 24h cells were harvested and stained in accordance to the manufacturer’s instructions. Viable, apoptotic and dead cells were measured by using the FL1-H and FL3-H channels. Data were analyzed using the WinMDI 2.8 software (Scripps Reserach Institute, La Jolla, CA, USA).

### Extraction of nuclear and cytoplasmic proteins

Extraction of nuclear and cytoplasmic proteins for NF-**κ**B Western Blot analysis was performed by using the NE-PER Nuclear and Cytoplasmic Extraction Reagents (Thermo Fisher Scientific, Bonn, Germany). Cells were harvested, resuspended (2×10^6^ cells) in cell culture media and treated with 100ng/ml LPS (Sigma-Aldrich, Taufkirchen, Germany) for 2h at 37°C and 5% CO_2_ in a humidified atmosphere. Non-stimulated cells served as a control. Nuclear and cytoplasmic proteins were extracted in accordance to the manufacturer’s instructions, boiled in 3× Laemmli Sample Buffer (5minutes, 95°C) [38] and stored at -80°C prior to SDS-PAGE and Western Blot analysis.

### Western blot analysis

Untreated or LPS-treated cells (2×10^5^) were harvested, washed once with PBS, and were lysed in 3× Laemmli Sample Buffer (5 minutes, 95°C) [38]. Samples were stored at -20°C before being separated on a 10% or 12%, respectively, SDS polyacrylamide gel and transferred to an Immobilon-P PVDF nitrocellulose membrane (EMD Millipore, Darmstadt, Germany) under semi-dry conditions. Membranes were blocked overnight with 10% (w/v) non-fat dry milk powder in TBS-T. The used antibodies are listed above. Bands were visualized using the Pierce ECL Western Blotting Substrate (Thermo Fisher Scientific, Bonn, Germany) in accordance to the manufacturer’s instructions and were detected with Aequoria Macroscopic Imaging System (Hamamatsu Photonics Germany, Herrsching am Ammersee, Germany). The relative expression levels of proteins in accordance to the housekeeping control were determined by using ImageJ (imagej.nih.gov/ij).

### ELISA

TNF-**α** expression was determined by using the human TNF-**α** ELISA Kit (Thermo Scientific, Bonn, Germany) in accordance to the manufacturer’s instructions. In brief, 1×10^5^ cells were cultivated in a 6-well plate and were treated for up to 6h with 100ng/ml LPS. After 2, 4, and 6h 75**μ**l supernatant was collected and was stored at -80°C. The supernatant of untreated cells served as a control. Microplates were analyzed using a BioTek Elx800^™^ microplate reader (BioTek, Bad Friedrichshall, Germany).

### Statistical analysis

Statistical significance was calculated using an unpaired, two-tailed Student’s *t*-test.

## Results

### TLR expression pattern in human MDA-MB-435-Hyg breast cancer cells, human M13SV1-EGFP-Neo breast epithelial cells, and M13MDA435-1 and -3 hybrids

**Fig 1:**
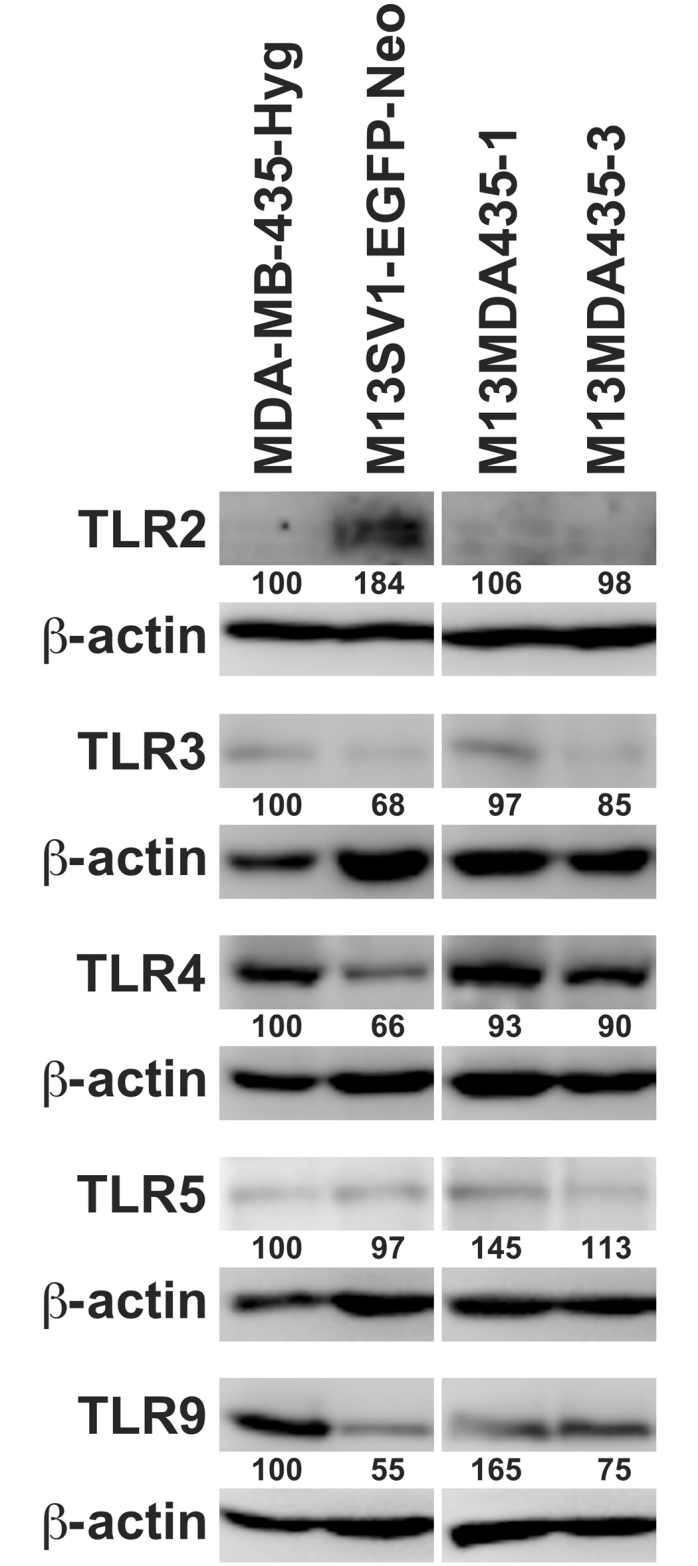
TLR expression pattern. Western Blot analysis revealed a marked expression of TLR4 and TLR9, whereas both TLR3 and TLR5 were rather moderately expressed in all cell lines. Interestingly, TLR2 expression was solely detected in M13SV1-EGFP-Neo breast epithelial cells. Shown are representative data of at least three independent experiments. The relative TLR expression levels of the cells were calculated in relation to the appropriate **β**-actin expression level, whereby MDA-MB-435-Hyg cells were set to 100%.

TLR expression of human MDA-MB-435-Hyg breast cancer cells, human M13SV1-EGFP-Neo epithelial cells, and M13MDA435-1 and -3 hybrid cell clones, which originated from spontaneous cell fusion events [23, 33], was analyzed by RT-PCR (S1 Fig) and Western Blot analysis (Fig 1). TLR4 and TLR9 were highly expressed, whereas only a moderate to weak TLR3 and TLR5 expression was detected in all cell lines (Fig 1, S1 Fig). Interestingly, TLR2 expression was solely detected in M13SV1-EGFP-Neo breast epithelial cells (Fig 1, S1 Fig).

### Proliferation of M13MDA435-1 and -3 hybrid cells, but not parental cells, is impaired by LPS

**Fig 2:**
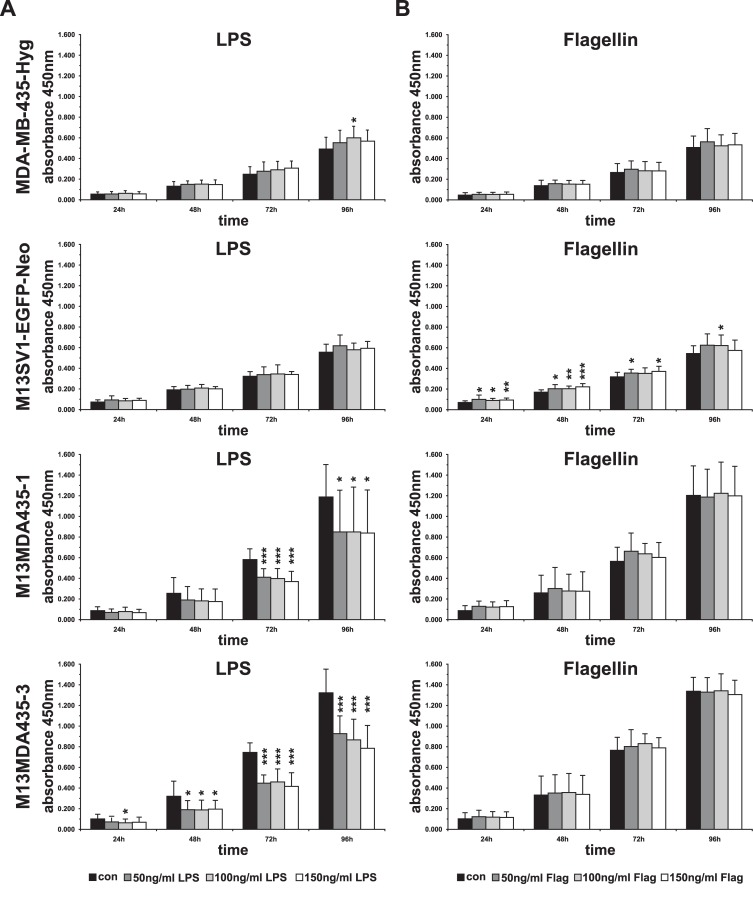
Cell proliferation data. Cell proliferation with the presence of different concentrations of LPS (**A**) and Flaggelin (Flag) (**B**). Data clearly show that LPS significantly impaired the proliferation of M13MDA435-1 and M13MDA435-3 hybrid cell lines, but not parental cell lines. Flaggelin served as a control. Shown are the mean ± STD of three independent experiments. Significance: * = p<0.05, ** = p<0.01, *** = p<0.001.

Because several studies implicated a correlation between TLR4 expression and breast cancer progression, including proliferation and invasiveness [10, 11, 37], we decided to perform initial cell proliferation studies by cultivating the cells in the presence of different concentrations of the TLR4 ligand LPS. Interestingly, and irrespective of the applied concentration, LPS significantly impaired the proliferation of M13MDA435-1 and -3 hybrid cells, whereas the cell growth of parental cells remained unaltered (Fig 2A). Because TLR5 and TLR4 exhibit a similar signal transduction cascade [39] control cell proliferation studies were performed within the presence of different concentrations of the TLR5 ligand Flagellin. In brief, proliferation of cells was not impaired by Flagellin (Fig 2B) suggesting a LPS specific effect.

### Induction of apoptosis in M13MDA435-1 and -3 hybrid cell lines by LPS

**Fig 3:**
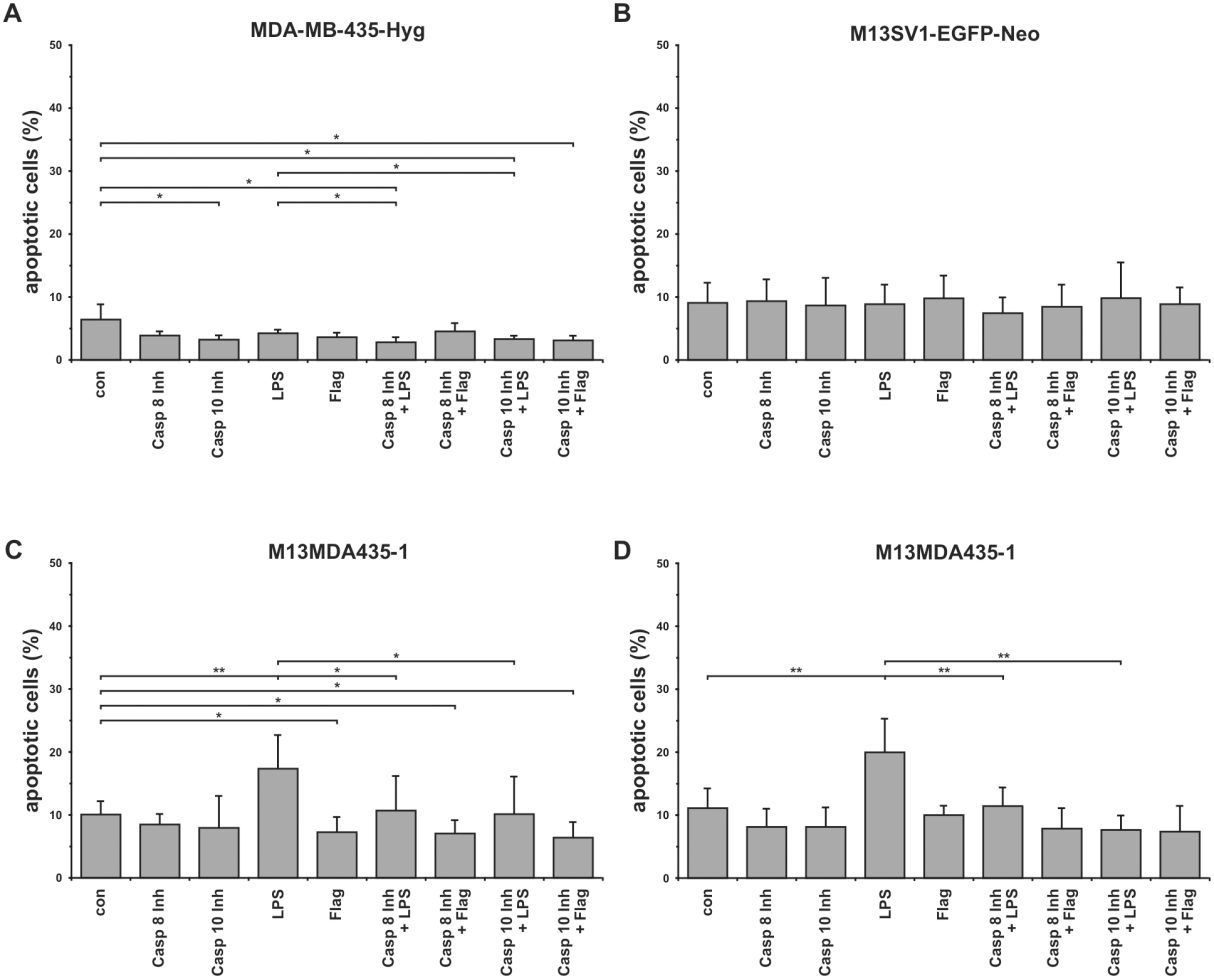
Induction of apoptosis in M13MDA435-1 and -3 hybrid cell lines by LPS. **A**) MDA-MB-435-Hyg, **B**) M13SV1-EGFP-Neo, **C**) M13MDA435-1, **D**) M13MDA435-3. LPS, Flagellin (Flag): 100ng/ml, Caspase 8 Inhibitor (Casp 8 Inh; Z-IETD-FMK; 10**μ**M), Caspase 10 inhibitor (Casp 10 Inh; Z-AEVD-FMK; 10**μ**M) Shown are the mean of at least three independent experiments. Significance: * = p<0.05, ** = p<0.01, *** = p<0.001.

LPS is a well-known inducer of apoptosis in e.g., endothelial cells and macrophages [40, 41]. To analyze whether the LPS impaired proliferation of M13MDA435-1 and -3 hybrid cell lines was attributed to apoptosis flow cytometry based apoptosis measurements were conducted. Cultivation of hybrid cells for 24h in the presence of 100ng/ml LPS resulted in a significantly increased amount of apoptotic cells (Fig 3C and 3D), which was potently blocked in the presence of specific caspase-8 (Z-IETD-FMK; 10**μ**M) and caspase-10 (Z-AEVD-FMK; 10**μ**M) inhibitors (Fig 3C and 3D). On the contrary, no pro-apoptotic effect of LPS was observed in parental cells (Fig 3A and 3B).

### LPS treatment resulted in NF-κB nuclear translocation and expression of TNF-α and IFN-β in M13MDA435-1 and -3 hybrid cells

**Fig 4:**
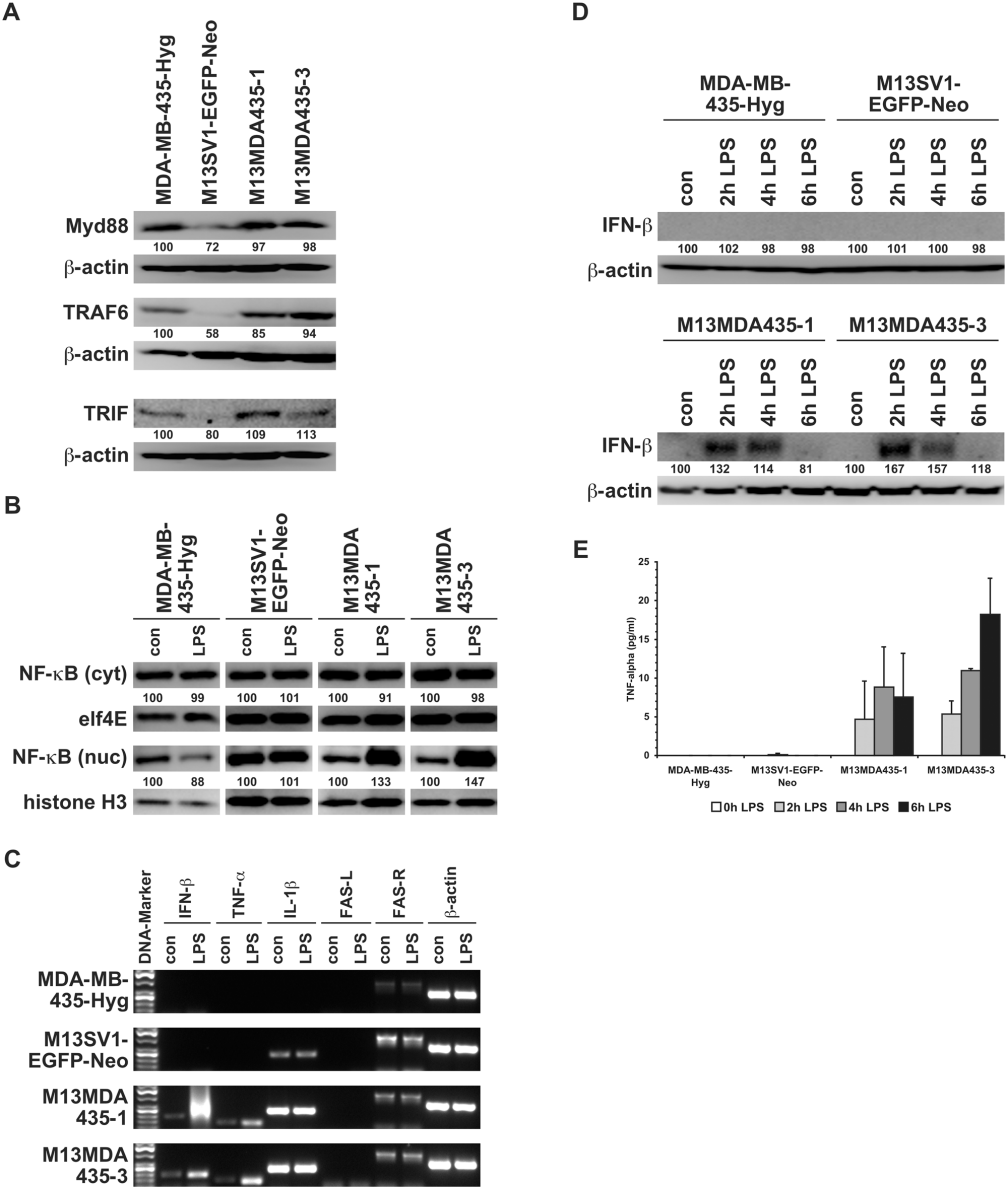
LPS treatment results in nuclear translocation of NF-κB and expression of NF-κB target genes in M13MDA435-1 and -3 hybrid cells. **A**) In comparison to MDA-MB-435-Hyg human breast cancer cells and M13MDA4351 and -3 hybrid cells lower expression levels of Myd88, TRAF6 and TRIF were detected in M13SV1-EGFP-Neo human breast epithelial cells. The relative protein expression levels were calculated in relation to the appropriate **β**-actin expression level, whereby MDA-MB-435-Hyg cells were set to 100%. **B**) A nuclear translocation of NF-**κ**B was only observed in LPS (100ng/ml, 2h) treated M13MDA-435-1 and -3 hybrid cells, but not parental M13SV1-EGFP-Neo and MDA-MB-435-Hyg cells. The relative protein expression levels were calculated in relation to the appropriate elf4E and histone H3 expression level. Controls were set to 100%. **C)** RT-PCR was performed to analyze the expression of LPS (IFN-**β**, TNF-**α**, IL-1**β**) and apoptosis related target genes (FAS-L, FAS-R) in untreated and LPS (100ng/ml, 2h) treated cells. A marked up-regulation of IFN-**β** and TNF-**α** was detected in M13MDA43-1 and -3 hybrid cells, but not parental cells. **D)** Validation of IFN-**β** up-regulation in M13MDA435-1 and -3 hybrid cells by Western Blot analysis. Cells were treated for up to 6h with 100ng/ml LPS. Shown are representative data of three independent experiments. The relative IFN-**β** expression levels were calculated in relation to the appropriate **β**-actin expression level. Controls were set to 100%. **E**) ELISA measurements confirmed a slight TNF-**α** up-regulation in M13MDA435-1 and -3 hybrid cells upon LPS treatment (100ng/ml, up to 6 h). Shown are the mean of three independent experiments.

LPS stimulation of TLR4 results in the induction of the myeloid differentiation factor 88 (Myd88) and the Toll/interleukin-1 receptor (TIR) domain-containing adaptor inducing IFN-**β** (TRIF) signal transduction pathways [1]. The Myd88-dependent pathway ultimately leads to NF-**κ**B nuclear translocation and expression of pro-inflammatory cytokines, whereas the TRIF-dependent pathway causes IRF3 nuclear translocation concomitant with expression of type 1 interferons [1]. We thus conducted Western Blot studies to investigate the expression levels of members of the Myd88 pathway (Myd88 and TRAF6) and the TRIF pathway (TRIF). Data are summarized in Fig 4A clearly showing that the expression levels of Myd88, TRAF6 and TRIF were markedly lower in M13SV1-EGFP-Neo breast epithelial cells as compared to MDA-MB-435-Hyg breast cancer cells and M13MDA435-1 and -3 hybrid cells (Fig 4A), which is further in accordance that a nuclear translocation of NF-**κ**B was not observed in LPS treated (100ng/ml, 2h) M13SV1-EGFP-Neo breast epithelial cells (Fig 4B). On the contrary, a strong LPS induced nuclear translocation of NF-**κ**B was observed in M13MDA435-1 and -3 hybrid cells (Fig 4B). Interestingly, LPS treatment did not result in an increased nuclear translocation of NF-**κ**B in MDA-MB-435-Hyg breast cancer cells despite Myd88 and TRAF6 expression (Fig 4B).

Analysis of IFN-**β**, TNF-**α**, IL-1**β**, FAS-L and FAS-R mRNA expression by RT-PCR in LPS treated and untreated cells revealed (a partially markedly) up-regulation of both IFN-**β** and TNF-**α** in LPS treated hybrid cell lines, but not parental cells (Fig 4C). RT-PCR data were confirmed by Western Blot analysis and ELISA, respectively, showing a peak level of IFN-**β** expression after 2 to 4h of LPS stimulation (Fig 4D) and rather low TNF-**α** expression levels in both hybrid cell lines (Fig 4E).

### LPS induced apoptosis in M13MDA435-1 and -3 hybrid cells is significantly impaired by neutralization of IFN-β, but not TNF-α

**Fig 5:**
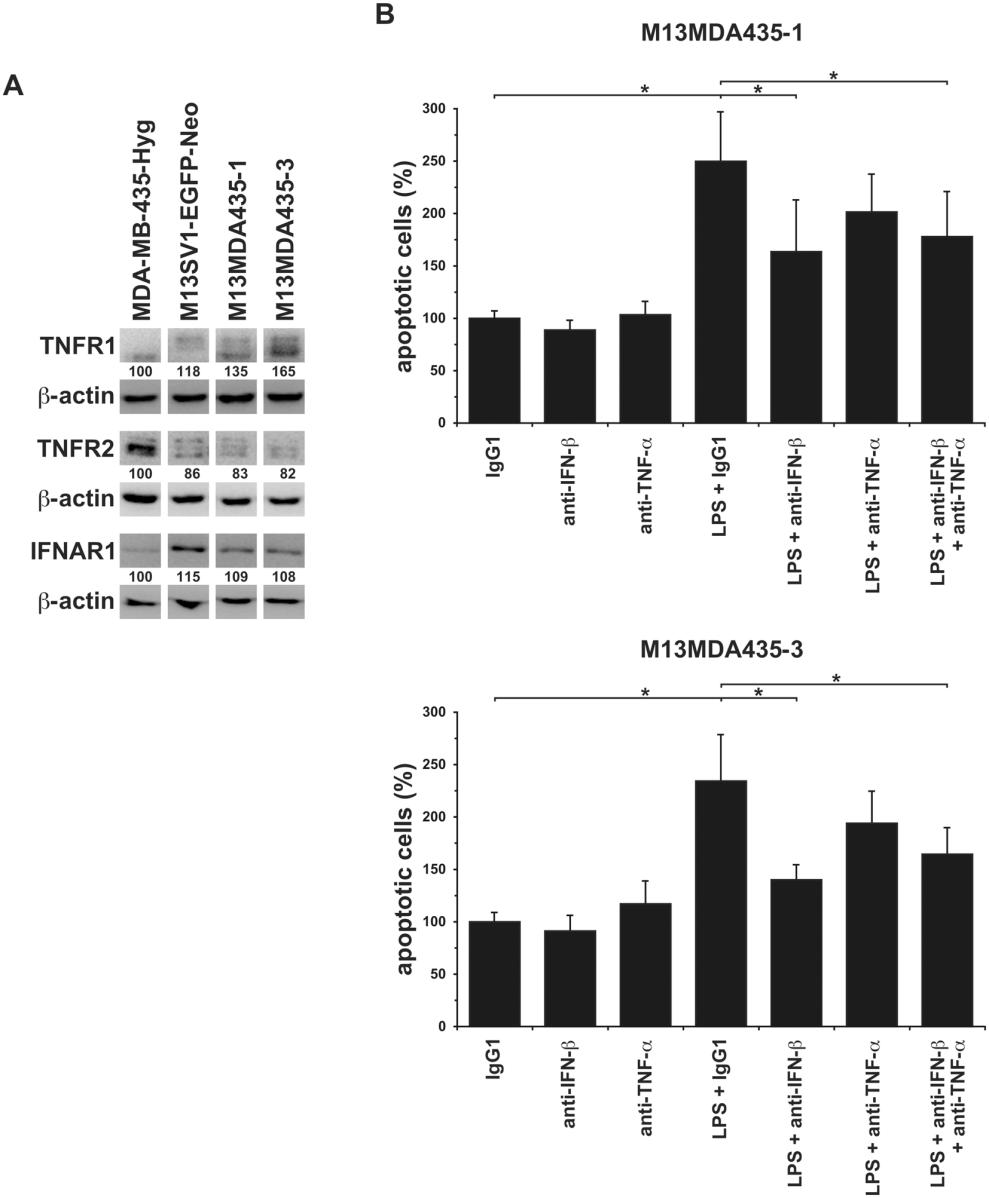
Neutralization of IFN-β, but not TNF-α, impaired the LPS induced apoptosis in M13MDA435-1 and -3 hybrid cells. **A)** Western Blot analysis revealed comparable TNFR1 and IFNAR1 expression levels in the investigated cell lines. By contrast, TNFR2 expression levels were markedly lower in M13SV1-EGFP-Neo human breast epithelial cells and M13MDA435-1 and -3 hybrid cells. Shown are representative data of three independent experiments. The relative protein expression levels were calculated in relation to the appropriate **β**-actin expression level, whereby MDA-MB-435-Hyg cells were set to 100%. **B**) M13MDA435-1 and -3 hybrid cells were cultivated in the presence of LPS (100ng/ml) and neutralizing IFN-**β** and TNF-**α** antibodies (10**μ**g/ml) for 24h. The relative amount of apoptotic cells was calculated in relation to the IgG1 control, which was set to 100%. Shown are the mean ± S.E.M. of five independent experiments. Significance: * = p<0.05.

Western Blot analysis revealed that both hybrid cell lines were positive for TNFR1 and IFNAR1 expression, whereas TNFR2 expression levels were rather low (Fig 5A). Cultivation of hybrid cell lines for 24h in the presence of different concentrations of IFN-**β** and TNF-**α**, respectively, revealed that both compounds were capable to induce apoptosis (S2 Fig). However, addition of neutralizing IFN-**β** and TNF-**α** antibodies showed that the LPS induced apoptosis in both hybrid cell lines was solely significantly impaired in the presence of an IFN-**β** blocking antibody (Fig 5B). A slight, but not significant impaired induction of apoptosis was observed for neutralization of TNF-**α** (Fig 5B). Because the level of inhibition of apoptosis mediated by IFN-**β** neutralization was comparable to when both IFN-**β** and TNF-**α** neutralizing antibodies were added we conclude that induction of apoptosis in M13MDA435-1 and -3 hybrid cells was chiefly induced by IFN-**β**. Nonetheless, a weak pro-apoptotic TNF-**α** effect can not be ruled out.

A siRNA-based approach to knock-down TNFR1/2 and IFNAR1 expression was not conducted since transfection techniques/reagents, such as nucleofection, Dharmafect and Lipofectin, markedly impaired the cells viability (S3 Fig).

### Induction of apoptosis in M13MDA435 hybrid clones is mediated via different mechanisms

To investigate whether LPS could also induce apoptosis in other hybrid cells two additional M13MDA435 hybrid clones (clone 2 and clone 4) were investigated, which also originated from spontaneous fusion events between human M13SV1-EGFP-Neo breast epithelial cells and human MDA-MB-435-Hyg breast cancer cells [23, 33]. In accordance to M13MDA435-1 and -3 hybrid cells (Fig 4A) clone 2 and clone 4 hybrid cells do also express TLR4, Myd88, TRAF6 and TRIF (S4 Fig). Conjointly, stimulation of M13MDA435-2 and -4 hybrid cells with 100ng/ml LPS for 2h was correlated with nuclear translocation of NF-**κ**B (S4 Fig), which is further in accordance to clone 1 and clone 3 data (Fig 4B). Interestingly, treatment of M13MDA435-2 and -4 hybrid cells with 100ng/ml LPS for up to 6h only induced a transient TNF-**α** expression, but not IFN-**β** (S4 Fig). Whether this was attributed to lower TRIF levels in clone 2 and 4 cells as compared to M13MDA435-1 and -3 hybrid cells remains unclear. In any case, LPS potently induced apoptosis in M13MDA435-2 and -4 hybrid cells (S4 Fig). In accordance to the lack of LPS-induced IFN-**β** expression addition of a IFN-**β** neutralizing antibody did not inhibit the LPS caused apoptosis (S4 Fig). This does also account for TNF-**α** neutralization even though clone 2 and clone 4 hybrid cells did express TNF-**α** in response to LPS stimulation (S4 Fig).

## Discussion

In the present study the role of TLR4 signaling in human M13SV1-EGFP-Neo breast epithelial cells, human MDA-MB-435-Hyg breast cancer cells and M13MDA453 hybrid cell lines was investigated. The rationale of this study was attributed to findings revealing an impact of TLR signaling in cancer [10, 11, 37] as well as the overall association of cell fusion in cancer, e.g., in the evolution of more malignant tumor (hybrid) cells [16, 19, 26, 27, 42]. However, in contrast to findings implicating a role of TLR4 in tumor progression our data rather indicate that TLR4 signaling potently induced apoptosis in M13MDA435 hybrid cells via different mechanisms. In M13MDA435-1 and -3 hybrid cells LPS mediated apoptosis is most likely induced via IFN-**β**, whereas in M13MDA435-2 and -4 hybrid cells the mechanism how LPS caused apoptosis remains unclear.

The finding that M13MDA435 hybrid cells exhibit a differential phenotype in comparison to their parental cells is in view with the cell fusion hypothesis [25, 26, 29]. Even though most studies investigating cell fusion in a cancer context are chiefly focusing on hybrid cell characteristics that could be linked to tumor progression it should be kept in mind that cell fusion is a random process and that the ultimate phenotype of the emerging hybrid cells can not be predicted. This matter is attributed to the heterokaryon-to-synkaryon transition [43] representing the merging of the parental nuclei to an unique hybrid cell nucleus—a process, which is commonly associated with chromosomal aberrations including deletions, translocation, amplifications, loss of whole chromosomes and putatively chromothripsis [25, 26, 29, 43–45]. Because this process runs in a unique and random manner, multiple discrete hybrid cell clones will originate, which (could) vary among each other and in relation to the parental cells. The diversity of various hybrid cells, which originated from weakly malignant Cloudman S91 melanoma cells and macrophages, was already demonstrated by Rachkovsky and colleagues [20]. Most of the hybrid cell clones possessed an increased metastatogenic capacity, thus forming faster and more metastases in shorter time than the parental melanoma cell line [20]. However, a few hybrid cell clones exhibited a decreased capacity of inducing metastases and three hybrid cell clones even failed to induce secondary lesions [20]. These findings are in view with recent data of Zhou et al. demonstrating that fusion of non-transformed rat intestinal epithelial cells gave rise to highly tumorigenic and non-tumorigenic hybrid cell clones [44].

The findings presented here show that LPS potently induced apoptosis via TLR4 signaling in M13MDA435 hybrid cell clones, but not in the parental cells. It is well recognized that LPS could also signal via TLR2 [46], but Western Blot data revealed that only M13SV1-EGFP-Neo breast epithelial cells were TLR2 positive, which, however, did not respond to LPS. Analysis of members of the TLR4 signal transduction cascade revealed comparable expression levels of Myd88 and TRAF6 in MDA-MB-435-Hyg breast cancer cells and M13MDA435 hybrid cells, whereas in M13SV1-EGFP-Neo breast epithelial cells the expression levels of these proteins were much lower. Likewise, a rather faint TRIF expression was detected in M13SV1-EGFP-Neo breast epithelial cells, which is opposite to the other cells exhibiting markedly higher TRIF expression levels. Several studies demonstrated the necessity of a functional Myd88 and TRIF signaling in mediating the cellular response to LPS [47–50] suggesting that the low expression levels of both proteins (and TRAF6) might be an explanation for the finding that NF-**κ**B target genes were not expressed in M13SV1-EGFP-Neo breast epithelial cells upon LPS stimulation. However, it can not be ruled out that the non-expression of TNF-**α** or IFN-**β** might be attributed to another mechanism, e.g., mediated by miRNA, since nuclear levels of NF-**κ**B were detectable in untreated and LPS treated cells. This may also account for MDA-MB-435-Hyg breast cancer cells, which, despite clear Myd88, TRAF6 and TRIF expression levels and NF-**κ**B nuclear localization did not show expression of NF-**κ**B target genes. Data of Nakshatri and colleagues indicated that NF-**κ**B is constitutively activated in MDA-MB-435 breast cancer cells due to low expression levels of the inhibitory proteins I**κ**B-**α**, I**κ**B-**β** and I**κ**B-**γ** [51]. Because of that we conclude that MDA-MB-435-Hyg breast cancer cells should possess a functional TLR4 signaling, but that the lack of expression of NF-**κ**B target genes might be attributed to an epigenetic and/or miRNA dependent mechanism.

Interestingly, LPS stimulation of M13MDA435-2 and -4 hybrid cells solely resulted in the expression of TNF-**α**, but not IFN-**β**, which is opposite to M13MDA435-1 and -3 hybrid cells. All M13MDA435 hybrid cells express comparable levels of Myd88, TRAF6 and TRIF. However, it can not be ruled that those mechanisms (epigenetic and/ or miRNA dependent) that prevent IFN-**β** expression in MDA-MB-435-Hyg breast cancer cells might be also active in M13MDA435-2 and -4 hybrid cells. As mentioned above, cell fusion is a random process resulting in the evolution of unique hybrid cell clones exhibiting a unique transcriptome, which does not only have an impact of the phenotype of the hybrid cells, but also on the hybrid cells signal transduction cascades and their kinetics. In this context we have already demonstrated that M13MDA435-1 and M13MDA435-3 hybrid cells exhibit a differential RAF-AKT cross-talk [24]. Analysis of RAF-1 S259 phosphorylation, being a major mediator of the negative regulation of RAF-1 by AKT, showed decreased pRAF-1 S259 levels in Ly294002 treated M13MDA435-1 hybrid cells, whereas pRAF-1 S259 levels remained unaltered in M13MDA435-3 hybrid cells [24]. Thus, inhibition of PI3K/AKT signaling by relieved the AKT mediated phosphorylation of RAF-1, which was accompanied with detection of phosphorylated MAPK in Ly294002 treated M13MDA435-1 hybrid cells [24]. Moreover, inhibition of the RAF-AKT cross-talk in M13MDA435-1 hybrid cells by Ly294002 was associated with an increased migratory activity of the cells [24] indicating the impact of cell fusion in giving rise to unique hybrid cells exhibiting differentially regulated signal transduction cascades and cross-talks.

Even though LPS potently induced apoptosis in all M13MDA435 hybrid cells it remains ambiguous why induction of apoptosis in M13MDA435-1 and -3 hybrid cells was most likely IFN-**β** dependent, whereas in M13MDA435-2 and -4 hybrid cells induction of apoptosis was IFN-**β** independent. The finding that LPS induced apoptosis in an IFN-**β** dependent manner in M13MDA435-1 and -3 hybrid cells is in view with data of Jung et al. demonstrating that the LPS mediated apoptosis of microglia cells depends on TLR4 initiated Myd88 and TRIF signal transduction pathways [52]. TRIF/IRF3 signaling induces IFN-**β** expression, which in turn initiates STAT1 signaling and induction of inducible NO synthase (iNOS) concomitant with production of apoptogenic NO [52]. Myd88 signaling caused NF-**κ**B activation, which not only induced expression of pro-inflammatory cytokines, but also expression of the caspase-11 and induction of caspase-3, but not caspase-8, mediated apoptosis [52]. Thus, both TLR4 induced signal transduction pathways synergistically induce apoptosis in microglia cells. However, because caspase-8 was neither activated by LPS induced TLR4 signaling including caspase-11 activation nor by IFN-**β** signaling in this study [52] we conclude that the IFN-**β** induced apoptosis in M13MDA435-1 and -3 hybrid cells must be mediated via a different mechanism. Here we have shown that the LPS induced apoptosis of M13MDA435-1 and -3 hybrid cells was caspase-8 and caspase-10 dependent.

Data of Chawla-Sarkar and colleagues provided evidence that IFN-**β** induced apoptosis in WM9 melanoma cells in a caspase-8 dependent manner via induction of an autocrine/paracrine TRAIL feedback loop [53]. Both, inhibition of caspase-8 activity using a specific inhibitor as well as using a TRAIL neutralizing antibody effectively impaired IFN-**β** mediated apoptosis in these cells [53]. Similar findings were reported for multiple myeloma cells where IFN-**β** induced apoptosis by TRAIL expression, which in turn signaled via an autocrine/ paracrine feedback loop through its receptors DR5 (or DR4) concomitant with recruitment of caspase-8 to the plasma membrane and subsequent cleavage [54]. Treatment of multiple myeloma cells with IFN-**β** also resulted in a decreased expression of the anti-apoptotic protein Bcl-XL, which might be an additional mechanism to shift the cells toward a pro-apoptotic state [54]. It would thus be of interest to investigate whether stimulation of hybrid cell lines with LPS or IFN-**β**, respectively, would result in TRAIL expression concomitant with a TRAIL dependent induction of apoptosis.

Studies on macrophages revealed that type I interferon signaling is a predominant mechanism of necroptosis since IFNAR1 deficient macrophages were highly resistant to necroptosis after stimulation with LPS or IFN-**β** in the presence of caspase inhibitors [55]. Thereby, IFNAR1 signaling eventually led to the assembly and activation of the ISGF3 complex, consisting of STAT1, STAT2, and IRF9, which in turn promoted a sustained activation of the Rip1/Rip3 necrosome complex concomitant with induction of necroptotic cell death [55]. Moreover, it is well recognized that also TLR4 signaling can contribute to necroptosis through a TRIF and Myd88 dependent mechanism [55]. On the contrary, necroptosis differs from apoptosis as the necrotic cell death program is independent on caspase activation [56]. Because LPS induced cell death was impaired in the presence of caspase inhibitors (caspase-8 and caspase-10) we conclude that LPS treatment rather induced apoptosis then necroptosis in M13MDA435-1 and -3 hybrid cells.

Various studies provided evidence that LPS could induce apoptosis via the intrinsic apoptosis pathway. Wang and colleagues demonstrated that LPS (in combination with cycloheximide) leads to the caspase-8 dependent cleavage of Bid in lung endothelial cells [57]. Bid activates Bax or Bak at the mitochondrial membrane, thereby causing the release of cytochrome c concomitant with induction of apoptosis [57]. Similar data were reported for the LPS induced apoptosis of tracheobronchial epithelial cells, which was also attributed to a LPS-dependent caspase-3 activation and cytochrome c release [58] indicating induction of the intrinsic apoptosis pathway. It would thus be of interest to investigate whether LPS may activate the intrinsic apoptosis pathway in M13MDA435-2 and -4 hybrid cells. LPS potently induced apoptosis in both hybrid clones in a caspase-8 dependent manner, but not via induction of IFN-**β** expression.

In summary, M13MDA435 hybrid cell lines differ from their parental cell lines by being susceptible to LPS-induced apoptosis. Thus our data are in view with the cell fusion hypothesis that hybrid cells could exhibit novel properties.

## Supporting Information

S1 Fig RT-PCR analysis of TLR expression. TLR expression pattern of the investigated cell lines was determined by RT-PCR. The used primer pairs are listed in Table 1. Shown are representative data of at least three independent experiments.
(EPS)

S2 Fig TNF-α and IFN-β are both capable to induce apoptosis in M13MDA435-1 and -3 hybrid cells. Cells were cultivated for 24h within different concentrations of TNF-**α** and IFN-**β** prior to determination of apoptotic cells by flow cytometry. TNF-**α** significantly induced apoptosis in both hybrid cell lines, but only when applied in a concentration of 100ng/ml. By contrast, a significant induction of apoptosis was achieved at any applied IFN-**β** concentration. Shown are the mean ± STD of the three independent experiments. Significance: * = p<0.05, ** = p<0.01, *** = p<0.001.
(EPS)

S3 Fig Impact of cell transfection procedures on the viability of M13MDA435-1 and -3 hybrid cells. Cells were transfected with the indicated techniques (Nucleofection; Lonza, Cologne, Germany) or transfection reagents (Dharmafect 1; GE Healthcare, Lafayette, CO, USA; Lipofectin; Thermo Fisher Scientific, Bonn, Germany) in accordance to the manufacturers’ instructions. After 24h apoptosis measurements cells were performed by flow cytometry. Shown are the mean ± STD of the three independent experiments.
(EPS)

S4 Fig LPS potently induce apoptosis in M13MDA435-2 and -4 hybrid cells, but in an IFN-β independent manner. **A)** Western Blot analysis of TLR4, Myd88, TRAF6 and TRIF. Shown are representative Western Blot data of at least three independent experiments. Protein expression was calculated in relation to **β**-actin. Expression levels of clone 2 were set to 100%. **B)** LPS treatment (100ng/ml, 2h) leads to nuclear translocation of NF-**κ**B in M13MDA435-2 and -4 hybrid cells. Shown are representative Western Blot data of at least three independent experiments. Protein expression was calculated in relation to **β**-actin or histone H3, respectively. Controls were set to 100%. **C)** Induction of a transient TNF-**α**, but not IFN-**β** expression in response to LPS stimulation (100ng/ml). Shown are representative Western Blot data of at least three independent experiments. Protein expression was calculated in relation to **β**-actin. Controls were set to 100%. **D)** M13MDA435-2 and -4 hybrid cells were cultivated in the presence of LPS (100ng/ml) and neutralizing IFN-**β** and TNF-**α** antibodies (10**μ**g/ml) for 24h. The relative amount of apoptotic cells was calculated in relation to the IgG1 control, which was set to 100%. Shown are the mean ± S.E.M. of three independent experiments. Significance: * = p<0.05. Data show that neither neutralization of TNF-**α** nor neutralization of IFN-**β** impaired the LPS induced apoptosis in M13MDA435-2 and -4 hybrid cells.
(TIFF)
