# Supplementary figures and images for: Lipopolysaccharide (LPS) Promotes Apoptosis in Human Breast Epithelial × Breast Cancer Hybrids, but Not in Parental Cells
Source: PLoS One. 2016 Feb 10;11(2):e0148438. doi: 10.1371/journal.pone.0148438 (PMC4749126; doi:10.1371/journal.pone.0148438)

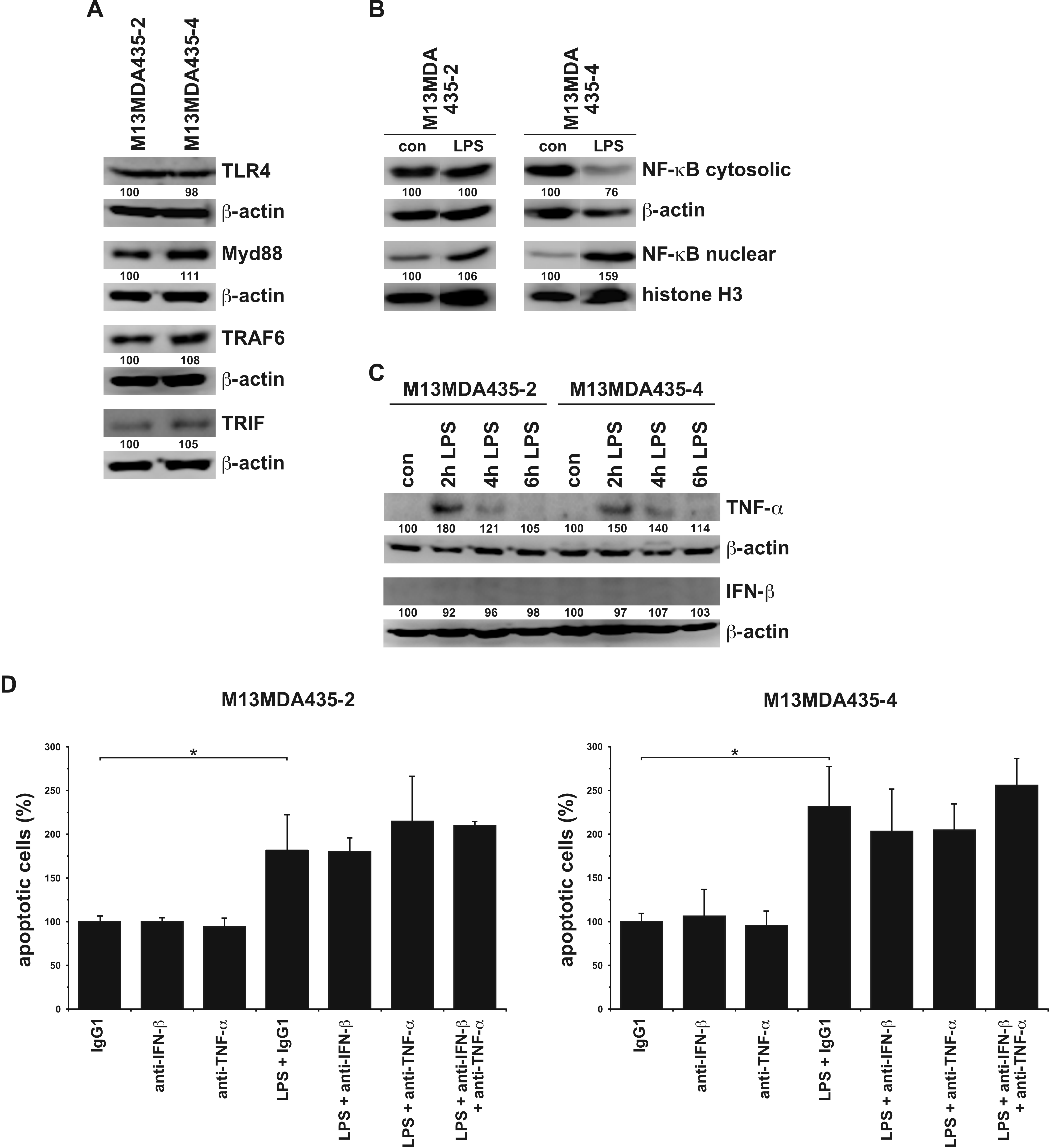

Supplement: S4 Fig — A) Western Blot analysis of TLR4, Myd88, TRAF6 and TRIF. Shown are representative Western Blot data of at least three independent experiments. Protein expression was calculated in relation to β-actin. Expression levels of clone 2 were set to 100%. B) LPS treatment (100ng/ml, 2h) leads to nuclear translocation of NF-κB in M13MDA435-2 and -4 hybrid cells. Shown are representative Western Blot data of at least three independent experiments. Protein expression was calculated in relation to β-actin or histone H3, respectively. Controls were set to 100%. C) Induction of a transient TNF-α, but not IFN-β expression in response to LPS stimulation (100ng/ml). Shown are representative Western Blot data of at least three independent experiments. Protein expression was calculated in relation to β-actin. Controls were set to 100%. D) M13MDA435-2 and -4 hybrid cells were cultivated in the presence of LPS (100ng/ml) and neutralizing IFN-β and TNF-α antibodies (10μg/ml) for 24h. The relative amount of apoptotic cells was calculated in relation to the IgG1 control, which was set to 100%. Shown are the mean ± S.E.M. of three independent experiments. Significance: * = p<0.05. Data show that neither neutralization of TNF-α nor neutralization of IFN-β impaired the LPS induced apoptosis in M13MDA435-2 and -4 hybrid cells. (TIFF) [file pone.0148438.s004.tiff]
